# Evaluation of health related quality of life in irritable bowel syndrome patients

**DOI:** 10.1186/1477-7525-10-12

**Published:** 2012-01-29

**Authors:** Raika Jamali, Arsia Jamali, Maryam Poorrahnama, Abdollah Omidi, Bardia Jamali, Neda Moslemi, Reza Ansari, Shahab Dolatshahi, Naser Ebrahimi Daryani

**Affiliations:** 1 Anatomical Sciences Research Center, Students' Scientific Research Center, Kashan University of Medical Sciences, Kashan, Iran; 2 Students' Scientific Research Center, Tehran University of Medical Sciences, Tehran, Iran; 3 Department of Clinical Psychiatry, Kashan University of Medical Sciences, Kashan, Iran; 4 Laser Research Center of Dentistry, Dental Research Center, Tehran University of Medical Sciences, Tehran, Iran; 5 Digestive Disease Research Institute, Tehran University of Medical Sciences, Tehran, Iran; 6 Department of Gastroenterology, Tehran University of Medical Sciences, Tehran, Iran

**Keywords:** Quality of life, Irritable bowel syndrome, Gender, Anxiety, Depression

## Abstract

**Background:**

Quality of life (QOL) is an important measure in the management of Irritable Bowel Syndrome (IBS). Controversy exists in the findings of studies evaluating QOL in IBS subtypes, and little is known about this issue in Iranian patients. Determination of the factors affecting QOL in IBS patients may influence treatment outcomes. The aims of this study are to: 1) compare QOL between subtypes in a sample of Iranian IBS patients, 2) determine the factors associated with QOL in IBS.

**Methods:**

This cross sectional study included two hundred and fifty IBS patients with the mean age (± standard deviation) of 31.62 (± 11.93) years that were referred to outpatient gastroenterology clinic. IBS patients were diagnosed based on Rome-3 criteria by a gastroenterologist, and then they were categorized into three subtypes according to the predominant type of bowel habit. The "QOL specific for IBS", "Stait-trait anxiety inventory", and "Beck depression inventory-2" questioners were used to evaluate QOL, anxiety, and depression symptoms, respectively.

**Results:**

The mean QOL scores in IBS mixed subtype (71.7 ± 25.57), constipation predominant subtype (80.28 ± 25.57), and diarrhea predominant subtype (76.43 ± 19.13) were not different. (P value: 0.05) In multivariate linear regression analysis, anxiety symptom scores were inversely correlated with QOL scores. [Standardized beta: -0.43, (95% confidence interval: -0.70, -0.39), P value: < 0.01]

**Conclusion:**

It seems reasonable to manage anxiety symptoms properly in IBS patients since this might increase their QOL.

## Background

Irritable Bowel Syndrome (IBS) is a common gastrointestinal disease [1]. It is associated with significant direct health-care costs and indirect costs related to impaired work productivity [2]. IBS patients are diagnosed based on Rome criteria and are categorized into different subtypes according to the predominant type of bowel habit [3].

IBS seems to have a great impact on the health related quality of life (QOL) of the patients [4]. The use of health related QOL assessments have come to interest for better understanding of the biopsychosocial model in the functional gastrointestinal disorders [5].

Controversy exists in results of previous studies about QOL in different IBS subtypes [6-11]. This finding might be related to differences in cultural (such as genetic, nutritional, and socio-demographical) and psychiatric co-morbidities (such as depression and generalized anxiety disorder) in the studied populations.

It seems reasonable to determine QOL in different IBS subtypes, and identify the factors affecting QOL in IBS patients, since these findings may influence treatment outcomes. While there are substantial data on comparison of QOL in IBS subtypes and factors independently associated with it in other countries, there is a paucity of literature on this issue in Iranian patients.

According to the result of previous studies some important variables that seemed to affect QOL in IBS patients (such as, depression and anxiety symptoms, self reported symptom severity, educational status, marital status, geographic distribution, age, gender, and IBS subtype) were selected and evaluated in this study [12-14].

This study was designed to: 1) compare QOL between IBS subtypes in a sample of Iranian patients, 2) determine the factors independently associated with QOL in these patients.

## Methods

### Ethical considerations

This study was performed according to the ethical standards for human experimentation. The clinical research committee of Kashan Shahid Beheshti Hospital approved the study protocol (No. 6173). After explaining the aim of the study, a written informed consent was obtained.

### Patients and methods

This cross sectional study was performed on consecutively selected IBS patients, older than 14 years, who referred to outpatient gastroenterology clinic of Kashan Shahid Beheshti Hospital between March and November 2010.

Patients with the following criteria were excluded from the study: 1) known organic gastrointestinal diseases, 2) history of abdominal trauma, surgery, or hospital admission for evaluation of abdominal pain, 3) exacerbation of bowel symptoms with consumption of milk or milk products, 4) alarm signs, 5) abnormal laboratory (hematological and biochemical profiles including thyroid function tests, anti tissue transglutaminase antibodies, stool exam, and urinalysis) findings, 6) abnormal findings in barium contrast radiographies, or in upper and lower gastrointestinal endoscopies. Alarm signs consist of new onset of symptoms at fifty years or older, weight loss, nocturnal diarrhea, anemia, dysphagia, bloody stools, and family history of cancer [3].

Upper Gastrointestinal endoscopy and colonoscopy with multiple duodenal and colon biopsies were performed by an expert board certified gastroenterologist for the evaluation of organic diseases including celiac disease and microscopic colitis. Gastrointestinal diseases were diagnosed based on the history, physical examination, laboratory data, imaging, and endoscopic studies that were performed by the same gastroenterologist. IBS and its subtypes were diagnosed according to Rome-3 criteria in the absence of alarm signs [3]. Those participants who were eligible for the study were included.

IBS with constipation (IBS-C) subtype was diagnosed if hard or lumpy stools were present in more than 25% and loose (mushy) or watery stools in less than 25% of bowel movements. IBS with diarrhea (IBS-D) subtype was diagnosed if hard or lumpy stools were present in less than 25% and loose (mushy) or watery stools in more than 25% of bowel movements. Mixed IBS (IBS-M) subtype was diagnosed if hard or lumpy stools in more than 25% and loose (mushy) or watery stools were present in more than 25% of bowel movements [3].

The consistency of stool was defined according to patient report, using Bristol form scale [15]. Hard or lumpy stools were defined if separate hard lumps like nuts (difficult to pass) or sausage shaped but lumpy stools were present. Loose (mushy) or watery stools were defined if fluffy pieces with ragged edges, a mushy stool or watery, no solid pieces, or liquid stools were present.

### Questionnaires

#### Health related quality of life questionnaire

Disease specific QOL for IBS (IBS-QOL) is a thirty four-item self-report questionnaire. This questionnaire is especially designed to evaluate QOL in IBS patients [16]. A five point Likert response scale (0 to 4) was used to measure how much each statement described the respondents' feelings (not at all, slightly, moderately, quite a bit, and extremely or a great deal). The questionnaire evaluated eight subscales: dysphoria, interference with activities, body image, health worry, food avoidance, social reaction, sexual function, and relationships [16]. All items scores were summed to calculate total scores. Higher total scores indicated better QOL.

#### Depression questionnaire

Presence and severity of the symptoms of depression in participants were determined using Beck Depression Inventory 2 [17]. This valid and reliable test is a twenty one-item self-report questionnaire that assesses the existence and severity of symptoms of depression (for screening and quick diagnosis) as listed in the American Psychiatric Association's Diagnostic and Statistical Manual of Mental Disorders Forth Edition (DSM-4) in adults and adolescence over thirteen years. The patients were asked to consider each statement as it was related to the way they felt in the past two weeks. Each item score ranges from zero to three. The total score that ranges from zero to sixty-three predicts the severity of depressive symptoms. The scores ranging from 0 to 13 are indicative of minimal depression, scores from 14 to 19 are considered to reflect a mild level of depression, scores from 20 to 28 are presenting moderate, and a score ranging from 29 to 63 is labeled severe [18].

#### Anxiety questionnaire

The presence and severity of symptoms of anxiety in participants were determined using Spielberger State/Trait Inventory [19]. This self-report questionnaire consists of 20 items for evaluation of state anxiety and 20 items for evaluation of trait anxiety. The trait anxiety assesses the general tendency for anxiety, while state anxiety is a measure of anxiety experienced at time of the test. Each item score ranges from one to four. All items scores in state and trait anxiety were summed to calculate total scores. The higher total scores indicated higher levels of anxiety symptoms. The scores ranging from 20 to 31 were indicative of mild state/trait anxiety symptom, scores from 32 to 53 were considered to reflect a moderate level of anxiety, and scores higher than 54 were labeled severe [19]. We used the sum of state and trait anxiety scores as the indicator of anxiety symptom. Therefore, the anxiety score ranged from 40 to 160 in this study.

The Persian version of the above mentioned questionnaires were shown to be valid and reliable for use by the previous studies in Iran [20,21]. The clinical psychiatrist supervised the participants while they were filling out the self-report questionnaires to ensure that they answered all questions. The participants could ask the clinical psychiatrist for clarifications regarding the meaning of the questions and then filled out the questionnaires themselves.

#### Self-reported symptom severity questionnaire

The global assessment of symptom severity in the past four weeks was measured using the question "How bad is the discomfort?" where discomfort was recognized as abdominal pain and IBS associated bowel symptoms. Responses were graded as mild (can be overlooked if I do not think about it), moderate (cannot be overlooked, but does not influence my lifestyle), or severe (influences my lifestyle).

#### Social characteristics

Educational status was categorized as "low degree" and "high degree" groups. The low degree group consisted of individuals who had education up to high school. The high degree group consisted of those who had education more than college. Marital status was categorized as "married" and "single" groups. The single group consisted of unmarried, widows, and divorced individuals. Geographic distribution was categorized into urban and rural. Residents of cities (in the area of study) for the past years were considered as the urban group. Those living in the countryside were considered as rural.

### Statistical analyses

The Kolmogorov-Smirnov test was used to evaluate the normal distribution of the continuous variables. Comparison of gender, self-reported symptom severity, educational status, marital status, and geographic distribution between IBS subtypes were performed using chi-squared test. Comparison of mean age, depression and anxiety symptoms, and QOL between IBS subtypes was performed by analysis of variances (ANOVA).

The correlation between QOL and age, gender, self-reported symptom severity, IBS subtype, educational status, marital status, and geographic distribution, anxiety and depression symptoms were analyzed using multivariate linear regression analysis and standardized beta were calculated.

The statistical analyses were performed using SPSS version 17 (SPSS, Chicago, IL, USA). The probability of the difference between the dependent and independent variables were considered significant if P value was less than 0.05.

## Results

Among the three hundred IBS patients visited in the outpatient gastroenterology clinic, two hundred and fifty patients (ranging from 14 to 61 years) with the mean (± Standard Deviation) age of 31.62 (± 11.93) years were eligible for participating in the study and were enrolled.

The QOL, anxiety and depression symptom scores were normally distributed (Z = 1.3, 1.4, and 1.2 respectively; all P values > 0.05).

The comparisons of mean (± Standard Deviation) age, QOL, anxiety and depression symptom, gender, self-reported symptom severity, educational status, marital status, and geographic distribution between IBS subtypes are demonstrated in table 1.

**Table 1:** Comparison of demographic and psychosocial characteristics between irritable bowel subtypes

	IBS-M N = 120	IBS-C N = 75	IBS-D N = 55	P value
**Age *(year)***	31.97 ± 13.46	32.90 ± 10.09	29.12 ± 10.42	0.18
**Gender *(number)***				
*Male*	60	22	39	0.35
*Female*	60	36	33	
**Quality of life**	71.70 ± 25.57	80.28 ± 25.57	76.43 ± 19.13	0.05
**Anxiety symptom**	91.42 ± 21.23	87.92 ± 19.95	92.90 ± 13.80	0.30
**Depression symptom**	14.49 ± 7.32	11.90 ± 8.39	14.25 ± 6.47	0.05
**Symptom severity**				
Mild	54	46	22	0.07
Moderate	47	22	27	
Severe	19	6	6	
**Educational status**				
Low degree	99	61	46	0.94
High degree	21	14	9	
**Marital status**				
Single	24	9	10	0.34
Married	96	66	45	
**Geographic distribution**				
Urban	113	70	50	0.72
Rural	7	5	5	

**IBS**, Irritable Bowel Syndrome; **M**, Mixed subtype; **C**, Constipation predominant subtype; **D**, Diarrhea subtype; **N**, Number

The relationship between QOL scores and age, gender, anxiety and depression symptom, self-reported symptom severity, educational status, marital status, geographic distribution, and IBS type in multivariate regression analysis are demonstrated in table 2.

**Table 2:** The relationship between quality of life and demographic and psychosocial characteristics in the study population

	Standardized beta	95% confidence interval for beta	P value
Age	0.08	(-0.43) - (0.07)	0.15
Gender	0.01	(-5.88) - (4.53)	0.79
Anxiety symptom	-0.43	(-0.70) - (-0.39)	< 0.01
Depression symptom	-0.06	(-0.69) - (0.29)	0.43
Symptom severity (mild versus others)	-0.12	(-13.21) - (0.85)	0.09
IBS subtype (M versus others)	-0.10	(-10.27) - (0.20)	0.06
Education status	-0.04	(-9.87) - (4.03)	0.41
Marital status	-0.00	(-8.17) - (7.66)	0.94
Geographic distribution	-0.08	(-18.94) - (1.85)	0.11

**IBS**, Irritable Bowel Syndrome; **M**, Mixed subtype

## Discussion

Based on the results of this study, QOL was not different between IBS subtypes. This finding is in consistence with the results of the previous studies that used different forms of generic and disease specific questionnaires for the evaluation of QOL [7-9,11,22-24]. However, in the study of Erikson et al QOL was lower in IBS-M and IBS-C when compared with IBS-D [7]. Meanwhile, Si et al reported that QOL in IBS-C is lower than IBS-D [10].

The anxiety and depression symptom mean scores were not different among IBS subtypes in this study. This is similar to the results of the previous studies that used different forms of questionnaires for the evaluation of anxiety and depression symptoms in IBS patients [8,9,23,25]. On the other hand, Erikson et al showed that the mean anxiety and depression scores were higher when IBS-C and IBS-M were compared with IBS-D using "Psychosocial Rating Scale" [7]. Meanwhile, Muscatello et al showed that the mean anxiety and depression scores were higher in IBS-C than IBS-D using "Anxiety Status Index" and "Hamilton Rating Scale for Depression" [8]. However, there was no significant difference in the mean anxiety and depression scores between IBS-C and IBS-D in the same study when "Hamilton Rating Scale for Anxiety" and "Montgomery-Asberg Depression Rating Scale" was used. Cho et al reported that IBS-C patients had more anxiety and depression symptoms than other subtypes using "Hospital Anxiety and Depression Scale" [24].

The comparisons of QOL, anxiety and depression symptoms in IBS subtypes increase our knowledge on the psychometric ability and clinical application of the used instruments. The differences among the results of above-mentioned studies on QOL, anxiety and depression symptoms between IBS subtypes may be related to the type of questionnaire used and cultural differences in the studied populations. When comparing the results of these cross sectional studies, we should keep in mind that IBS subtypes may change into one another if they are followed for long enough periods, therefore comparison of these parameters between IBS subtypes might change [9].

In this study, QOL was independently associated with anxiety; however, age, gender, depression symptom, IBS subtypes, symptom severity, geographic distribution, educational, and marital status were not related to QOL. Jerndal et al proposed that anxiety was an important factor affecting QOL in IBS patients [26]. Kanasawa et al found that QOL was not related to gender, IBS subtypes, educational, and marital status [27]. Simren et al reported that IBS subtypes did not affect QOL [28]. Another study, which used "World Health Organization QOL-BREF" and "Bradley well-being QOL" measures, showed that QOL was not affected by socio-demographic parameters in diabetic patients [29].

### Clinical application

As the result of this study showed, QOL was inversely correlated with anxiety symptom. Therefore, the proper management of anxiety symptom in IBS patients may improve their QOL.

### Strengths and limitations

The strength of this study was comparison of QOL considering anxiety and depression symptoms in an acceptable sample size of IBS patients. To recognize the IBS patients accurately, the gastroenterologist and not a general practitioner examined the participants. The clinical psychiatrist supervised and assisted the participants to complete the questionnaires precisely and completely.

One limitation of this study is evaluating just the outpatients. This study included IBS patients who referred to the gastroenterology clinic as an outpatient, and patients with a history of hospital admission for evaluation of abdominal pain were excluded. Therefore, the results of this study cannot be generalized to all IBS patients, especially those with severe symptoms who need hospitalization for further managements and even those with mild symptoms that do not consult a physician for their symptoms.

The use of cross sectional design is the second limitation of this study, since the changes of QOL after proper management could not be evaluated. Considering the conversion of subtypes into one another in the natural course of disease, this cross sectional study has a limited value for the evaluation of QOL, anxiety, and depression symptoms in IBS subtypes [10].

### Future work

Longitudinal prospective study to assess the changes of QOL after proper management, and evaluation of factors affecting QOL in Iranian IBS subtypes including those with severe symptoms (inpatients) and those with mild symptoms (non-consulters in general population) is recommended.

## Conclusions

This cross sectional study conducted on a sample of Iranian IBS patients referred to a secondary health care center showed that: 1) the mean QOL, anxiety and depression symptom scores were not different among subtypes. 2) QOL was inversely correlated with anxiety. Therefore, it seems reasonable to manage anxiety symptom in IBS patients to increase their QOL.

## List of abbreviations

IBS: Irritable bowel syndrome; IBS-C: Irritable bowel syndrome constipation predominant type; IBS-D: Irritable bowel syndrome diarrhea predominant type; IBS-M: Irritable bowel syndrome mixed type; IBS-QOL: Disease specific quality of life questionnaire for Irritable bowel syndrome; QOL: Health related quality of life.

## Competing interests

This study was supported by research funds of Kashan University of Medical Sciences.

## Authors' contributions

RJ, RA, SD, and ND made contributions to the design of the study. RJ performed the endoscopy and colonoscopy with mucosal biopsies, diagnosed IBS, and distinguished subtypes. RJ, AJ, MP, and AO participated in data collection. RJ, BJ, and NM, analyzed and interpreted the data. All authors read and approved the final manuscript.
